# A sensitive mass spectrometric assay for mitochondrial CoQ pool redox state *in vivo*

**DOI:** 10.1016/j.freeradbiomed.2019.11.028

**Published:** 2020-02-01

**Authors:** Nils Burger, Angela Logan, Tracy A. Prime, Amin Mottahedin, Stuart T. Caldwell, Thomas Krieg, Richard C. Hartley, Andrew M. James, Michael P. Murphy

**Affiliations:** a MRC Mitochondrial Biology Unit, University of Cambridge, Hills Road, Cambridge, CB2 0XY, UK; b Department of Medicine, University of Cambridge, Addenbrooke's Hospital, Cambridge, CB2 0QQ, UK; c Department of Physiology, Institute of Neuroscience and Physiology, Sahlgrenska Academy, University of Gothenburg, Gothenburg, Sweden; d School of Chemistry, University of Glasgow, Glasgow, G12 8QQ, UK

**Keywords:** CoQ_10_, CoQ_9_, Mass spectrometry, Redox state, Oxidative stress, Mitochondria, Coenzyme Q

## Abstract

Coenzyme Q (CoQ) is an essential cofactor, primarily found in the mitochondrial inner membrane where it functions as an electron carrier in the respiratory chain, and as a lipophilic antioxidant. The redox state of the CoQ pool is the ratio of its oxidised (ubiquinone) and reduced (ubiquinol) forms, and is a key indicator of mitochondrial bioenergetic and antioxidant status. However, the role of CoQ redox state *in vivo* is poorly understood, because determining its value is technically challenging due to redox changes during isolation, extraction and analysis. To address these problems, we have developed a sensitive liquid chromatography-tandem mass spectrometry (LC-MS/MS) assay that enables us to extract and analyse both the CoQ redox state and the magnitude of the CoQ pool with negligible changes to redox state from small amounts of tissue. This will enable the physiological and pathophysiological roles of the CoQ redox state to be investigated *in vivo*.

## Highlights

• Sensitive LC-MS/MS assay to quantify CoQ redox state.
• Changes in CoQ redox state can be measured in mitochondria, cells and *in vivo*.
• CoQ redox state is stable during extraction and analysis.

## Graphical abstract

**Figure:**
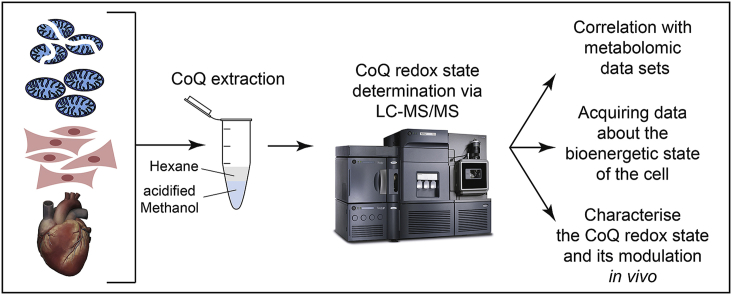

## 1 Introduction

Coenzyme Q (CoQ) is a hydrophobic respiratory chain component within the mitochondrial inner membrane that is also found to a far lesser extent in other membranes [1] (Fig. 1a). The final steps of CoQ biosynthesis take place in mitochondria, where the redox active benzoquinone headgroup is linked to an isoprenyl chain in which the number of isoprenoid units varies with species: in yeast it is CoQ_6_, in rodents it is mainly CoQ_9_ with some CoQ_10_, while in humans CoQ_10_ predominates over CoQ_9_ [[2], [3], [4], [5], [1]]. Within the mitochondrial inner membrane CoQ collects electrons from dehydrogenases, notably complexes I and II (Fig. 1a), during which the ubiquinone form, CoQ, undergoes two-electron reduction to ubiquinol (CoQH_2_) (Fig. 1b). The CoQH_2_ is then reoxidised to CoQ at complex III to enable the respiratory chain to reduce O_2_ to water while pumping protons across the inner membrane to establish the protonmotive force (Δp) that drives ATP synthesis by oxidative phosphorylation [1,6] (Fig. 1a). In addition to complexes I and II, a number of other dehydrogenases also feed electrons into the CoQ pool from diverse metabolic pathways [1,7] (Fig. 1c). As well as its role in the respiratory chain, CoQH_2_ is also an important antioxidant within the mitochondrial inner membrane where it acts as a chain breaking antioxidant to prevent lipid peroxidation [[2], [3], [4], [5], [1]].

**Fig. 1:**
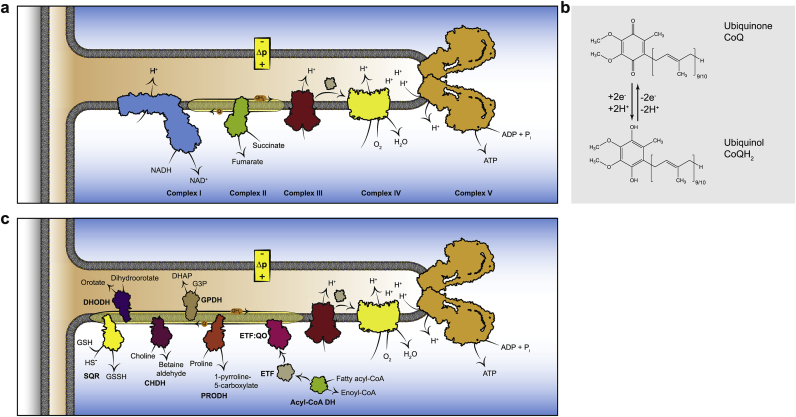
The CoQ pool in the mitochondrial inner membrane as essential link between metabolic pathways and cellular energy supply. Various mitochondrial enzymes of diverse metabolic function feed electrons into the CoQ pool of the inner mitochondrial membrane. The electrons are then relayed to complex III of the ETC and thereby coupled to the maintenance of Δp and ATP production. **a** Complex I (NADH:Ubiquinone oxidoreductase) and complex II (succinate dehydrogenase) of the ETC chain are the canonical enzymes feeding electrons into the CoQ pool under normal conditions. The electrons are shuttled to complex III (ubiquinol:cytochrome *c* oxidoreductase) to be transferred onto cytochrome *c* and finally oxidised by complex IV (cytochrome *c* oxidase). By pumping protons from the matrix into the intermembrane space, complexes I, III & IV establish the mitochondrial protonmotive force (Δp), which is used for ATP synthesis by complex V (ATP-synthase) and various transport processes across the inner membrane. **b** The two electron carrier ubiquinone is a 1,4-benzoquinone, linked to an isoprene tail which in mammals consists of 9–10 isoprenyl subunits. Ubiquinone is reduced to ubiquinol by two electrons and acts as electron shuttle and antioxidant. **c** Numerous other mitochondrial oxidoreductases, such as SQR (hydrogen sulfide:ubiquinone oxidoreductase, catabolism of hydrogen sulphide), DHODH (dihydroorotate dehydrogenase, pyrimidine biosynthesis), CHDH (choline dehydrogenase, choline oxidation), G3PDH (glycerol 3-phosphate dehydrogenase, glycerol-3-phosphate shuttle), ETF-QO (electron-transferring-flavoprotein dehydrogenase, fatty acid oxidation) and PRODH (proline dehydrogenase, catabolism of proline) feed electrons into the mitochondrial CoQ pool. The electrons are funnelled by CoQ via complex III and cytochrome *c* to complex IV to reduce O_2_ to H_2_O.

Thus, the CoQ pool plays a central role in mitochondrial function and is the organelle's principal point of contact with many other metabolic pathways. Consequently CoQ deficiency contributes to mitochondrial dysfunction, disease and ageing [[8], [9], [10]]. The redox state of the CoQ pool is the ratio of its reduced (CoQH_2_) and oxidised (CoQ) forms, and is a key indicator of mitochondrial bioenergetic and antioxidant status. The CoQ redox state alters dynamically in response to its relative rates of reduction by dehydrogenases and oxidation by complex III or by reactive oxygen species (ROS). Changes in the CoQ redox state are central to mitochondrial redox signalling in oxygen sensing [11] and inflammation [12,13] and also to the tissue damage associated with ischaemia reperfusion injury [[14], [15], [16]]. Therefore, the CoQ redox state is central to health and disease and measuring it *in vivo* is vital.

While assessing the size of the CoQ pool in tissues *in vivo* is straightforward, measuring its redox state *in vivo* is technically challenging. This is mainly due to the difficulty of stabilising the redox state of the CoQ pool during isolation, extraction and analysis. In addition, for many analytical methods large amounts of material are required, limiting applicability. Methods based on liquid chromatography coupled to electrochemical detection were used extensively in the past to investigate CoQ levels and the CoQ redox state in biological samples [[17], [18], [19], [20]]. More recently several LC-MS/MS approaches for analysis of the CoQ redox state have been described [[17], [18], [19], [20], [21], [22], [23]]. In these protocols single-phase CoQ extraction with relatively polar alcohols such as methanol or propanol are used [17,[20], [21], [22], [23]]. However, as the samples change redox state during isolation and storage, samples have to be analysed rapidly and in small batches. Recently, an improved protocol was developed for the determination of the CoQ redox state in tissues by extraction into non-polar hexane followed by analysis in acidified ethanol [18]. While this limits oxidation, separate standard curves were still required for the two redox states, with the CoQH_2_ standard curve being a potential limitation due to oxidation of CoQH_2_ standards distorting the CoQ redox state. Here we describe a simplified two-phase extraction that utilises a single internal standard (IS) that generates a stable extract in which both the redox state of the CoQ pool and its amount can be determined. This will facilitate the analysis of both *in vitro* and *in vivo* models and expand our understanding of the role of the CoQ pool in health and disease.

## 2 Results

### 2.1 CoQ LC-MS/MS assay

To establish CoQ redox state detection by LC-MS/MS, CoQ_9/10_, CoQ_9/10_H_2_ and *d*_*6*_-CoQ_10_ internal standard (IS) were prepared (Fig. 2a). Precursor scans of direct infusion of methanol/ammonium formate solutions of these compounds detected the H^+^ and NH_4_^+^ adducts that gave product ions at 197 *m/z* for both ubiquinone and ubiquinol, and at 203 *m/z* for the IS (Fig. 2b). We chose to use NH_4_^+^ adducts for LC-MS/MS analysis as they were more abundant, presumably due to higher concentrations of NH_4_^+^ compared to H^+^ within the buffer and because for both redox forms the H^+^ adducts showed the same mass for CoQ_9_ and CoQ_10_, making CoQ redox state analysis impossible. The proposed fragmentation mechanism is shown in Fig. S1. LC-MS/MS analysis of pure samples of each compound showed that the CoQ and CoQH_2_ were detected in the appropriate channel (Fig. 2c). While there was some bleed through of ubiquinone samples into the ubiquinol *m/z* channel, ubiquinone and ubiquinol were readily separated by LC (Fig. 2c). Hence, this LC-MS/MS analysis can easily separate the two redox forms, despite a mass difference of only 2.

**Fig. 2:**
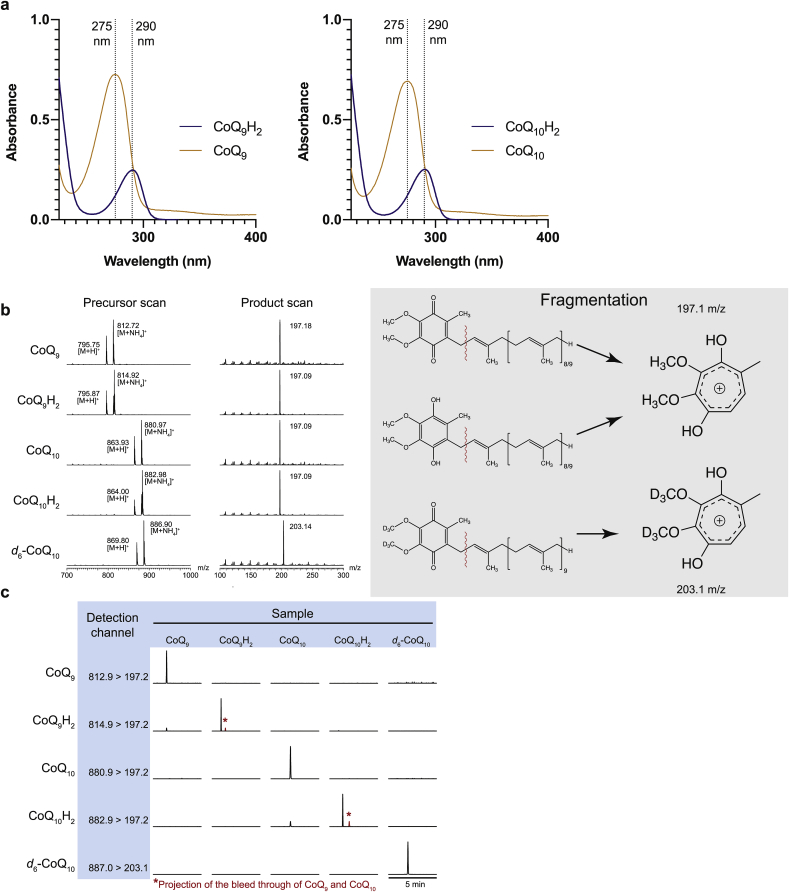
Method development for the detection of CoQ_9/10_ and CoQ_9/10_H_2_ by LC-MS/MS **a** The absorbance CoQ_9_ and CoQ_10_ were characterised by spectral scanning before and after reduction with NaBrH_4_. Upon reduction, the characteristic absorption maximum shifts from 275 to 290 nm **b** Representative precursor and product MS scans of CoQ_9_ and CoQ_10_ stocks and their reduced forms as well as the deuterated *d*_6_-CoQ_10_ internal standard. Precursor scans of the characteristic fragment products were performed. The product scans were performed by fragmenting the ammonium adduct precursors. Upon fragmentation the CoQ precursor loses its isoprene side chain and forms a tropylium ion. **c** Representative LC-MS/MS chromatograms showing the *m*/*z* transitions measured simultaneously for 0.5 pmol of CoQ_9_, CoQ_10_, CoQ_9_H_2_, CoQ_10_H_2_ and *d*_6_-CoQ_10_. Traces are normalised to the highest peak for each sample. CoQ_9_ and CoQ_10_ show bleed through into the transitions for CoQ_9_H_2_ and CoQ_10_H_2_, respectively. Bleed through of the CoQ_9_ and CoQ_10_ signals has been projected in red onto the CoQ_9_H_2_ and CoQ_10_H_2_ traces to show they are separated by LC. (For interpretation of the references to colour in this figure legend, the reader is referred to the Web version of this article.)

### 2.2 CoQ redox state determination

To determine the redox state of the CoQ pool it would be most convenient to measure the ratio of the mass spectrometer response to the two redox forms, to avoid having to construct standard curves for both redox forms of CoQ_9_ and CoQ_10_. For this to work the relative mass spectrometer response to CoQ and CoQH_2_ must be stable over a range of concentrations and ratios of both forms. To see if this was the case, the peak areas following analysis of equal amounts of CoQ and CoQH_2_ at a range of increasing total concentrations were compared (Fig. 3a). This analysis indicated that both redox forms of CoQ_9_ and CoQ_10_ had similar mass spectrometric responses. Furthermore, when samples containing a range of CoQH_2_/CoQ ratios were assessed the LC-MS/MS analysis also detected these ratios accurately (Fig. 3b). As the CoQH_2_/CoQ ratio also determines the % reduction of the CoQ pool, we replotted these data as % reduction (Fig. 3c) and also the reduction potential for the CoQ/CoQH_2_ redox couple [24] (Fig. 3d). Of note, for CoQH_2_/CoQ ratios of 100:1, in contrast to ratios ≤50:1, the measured value did not match that of the input sample, indicating that at very low CoQ reduction potentials some CoQH_2_ oxidation will inevitably occur. However, these highly reduced ratios are beyond the biologically relevant range. These analyses showed that measurement of the uncorrected ratio of the mass spectrometric response to both redox forms enables simultaneous and accurate determination of the redox states of both the CoQ_9_ and CoQ_10_ pools. We also wanted to investigate if CoQH_2_ has the potential to transfer electrons to CoQ following extraction, and thereby distort the measured ratios. To do this we mixed CoQ_9_H_2_ and CoQ_10,_ or CoQ_10_H_2_ and CoQ_9_, in methanol containing 2 mM ammonium formate and incubated for 2 h at 37°C (Figs. S2a and b). This showed some slow oxidation of CoQH_2_, but this was not associated with direct reduction of the ubiquinone of the other species (Figs. S2a and b). This analysis showed that the oxidation of the ubiquinols was slow under our conditions, even at 37°C, and that this oxidation was not associated with ubiquinone reduction. Therefore, during cold storage of the extracts oxidation of CoQH_2_ will have minimal effect on the CoQH_2_/CoQ ratio.

**Fig. 3:**
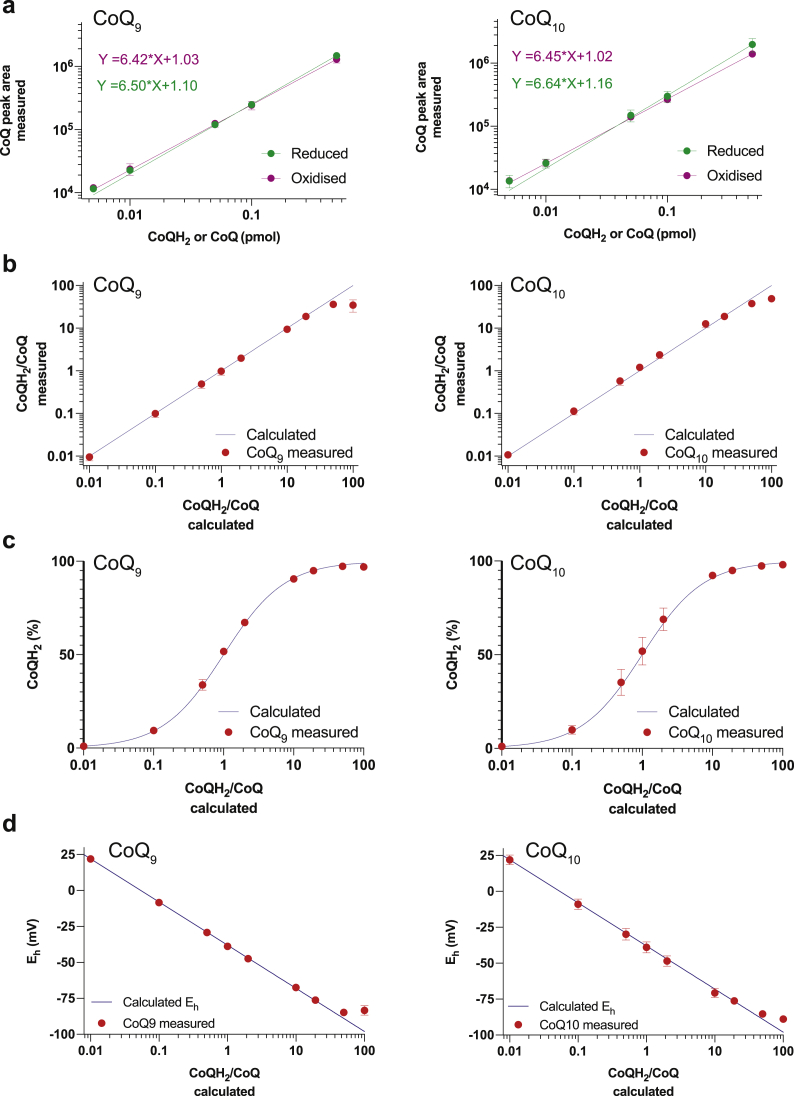
Determining and quantifying the ratio of CoQ and CoQH_2_ by LC-MS/MS To validate the quantification of oxidised and reduced CoQ, CoQ_9/10_ and borohydride reduced CoQ_9/10_H_2_ stocks were mixed in predetermined ratios and measured by LC-MS/MS. **a** Detected peak areas of increasing amounts of CoQH_2_ and CoQ mixed in a 1:1 ratio. **b** The detected CoQH_2_/CoQ ratios were blotted against the expected theoretical CoQH_2_/CoQ ratios. **c** The proportion of CoQH_2_ was blotted against the expected theoretical CoQH_2_/CoQ ratios. **d** The redox potential of CoQH_2_/CoQ mixtures was calculated based on the detected CoQH_2_/CoQ ratios and was blotted against the expected theoretical CoQH_2_/CoQ ratios. The calculations are outlined in the methods section. Data are mean ± S.D. of 3 replicates.

### 2.3 CoQ_9_ and CoQ_10_ quantification

The above analysis showed that the CoQH_2_/CoQ redox ratio could be measured without the requirement for standard curves, simplifying the analysis and removing potential confounding factors of ubiquinol oxidation during standard curve preparation. However, it is also important to determine the sizes of the CoQ_9_ and CoQ_10_ pools. To do this we used *d*_*6*_-CoQ_10_ as an IS which was synthesised by base-catalysed exchange of the methoxy groups of CoQ_10_ dissolved in hexane-CD_3_OD. This generated pure compound that could be used as an internal standard. The two-stage preparation described previously [19] proved unnecessary. Using *d*_*6*_-CoQ_10_ as IS we constructed standard curves for CoQ_9_ and CoQ_10_ (Fig. 4a and b). This enables the total amounts of CoQ_9_ and CoQ_10_ in a sample to be determined, in parallel with measurement of their redox ratios.

**Fig. 4:**
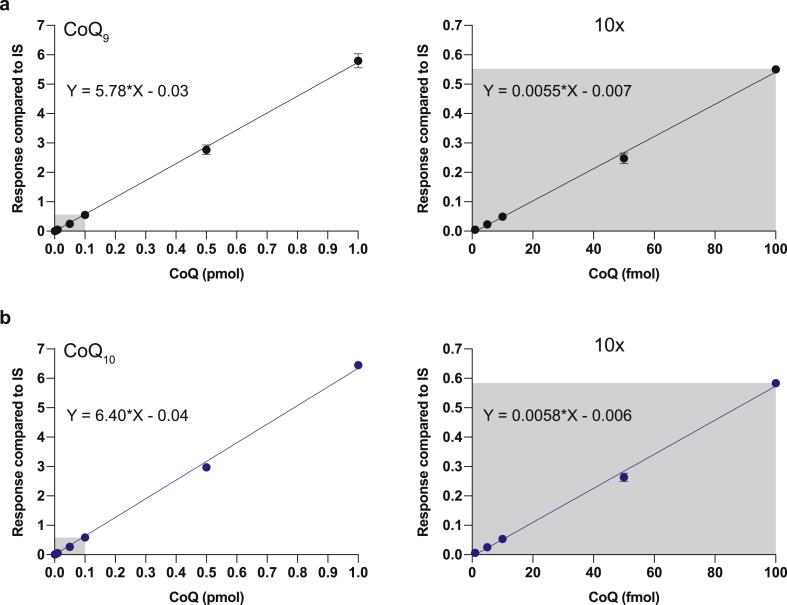
Standard curve for the quantification of the CoQ pool size by LC-MS/MS Representative standard curve or oxidised CoQ_9_ (**a**) and CoQ_10_ (**b**). The MS response of increasing concentrations of CoQ_9_ and CoQ_10_ was compared to 0.5 pmol of d_6_-CoQ10 (internal standard, IS). Data are mean ± S.D. of 3 replicates. The grey-shaded section of the standard curve on the left is expanded and replotted in the right panel.

### 2.4 Measurement of the CoQ redox state in mitochondria and cells

Next, we extended the CoQ extraction method to the analysis of isolated mitochondria and cells. For bovine heart mitochondrial membranes and isolated rat heart mitochondria we used a two-phase extraction with ice-cold hexane and acidified methanol (Fig. 5a). This precipitated proteins and retained many potential redox active polar metabolites in the aqueous-methanol phase, while acidification helped prevent oxidation of ubiquinol. Finally, extracting the CoQ pool into hexane generated a stable solution which was then processed for mass spectrometry.

**Fig. 5:**
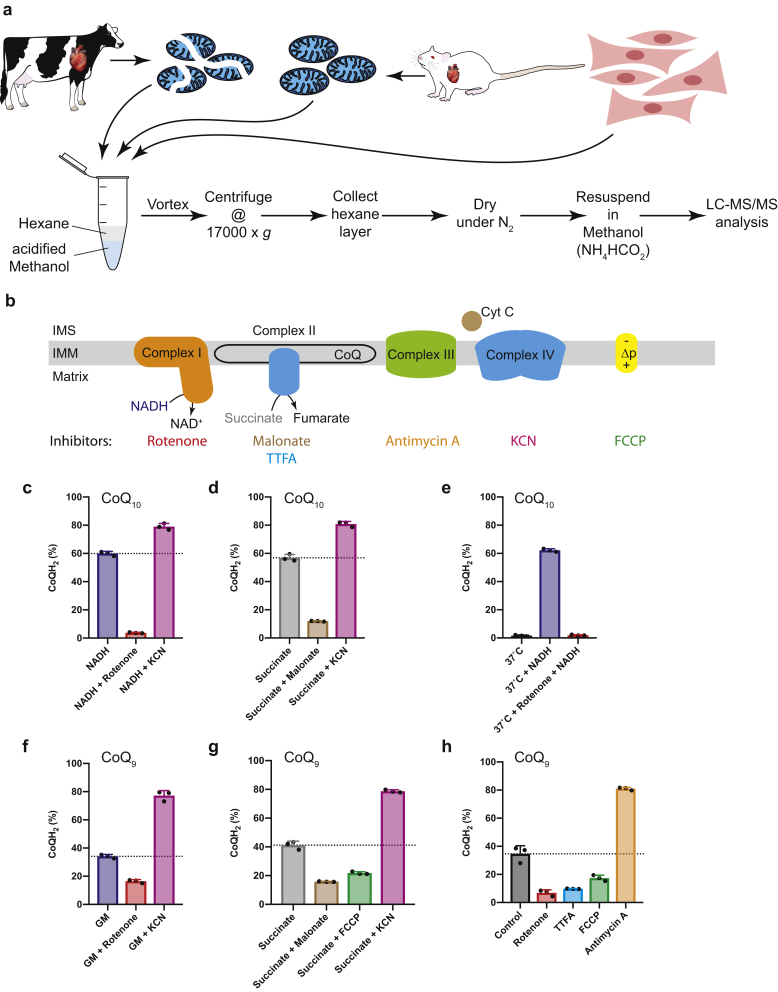
Extracting CoQ from biological samples and modifying the CoQ redox state in mitochondrial membranes, mitochondria and cells. **a** A two-phase extraction with acidified methanol and hexane was used to extract CoQ from bovine mitochondrial membranes, mitochondria and cells. CoQ from incubations of biological samples was extracted by vortexing in extraction solution, phase separation by centrifugation and resuspending the extract in MS sample buffer (methanol with 2 mM ammonium formate). **b** Various inhibitors can be used to manipulate the mitochondrial CoQ redox state in biological samples. **c-h** Bovine heart mitochondrial membranes (BHMM), rat heart mitochondria (RHM) and C2C12 cells were incubated in KPi (BHMM), KCl (RHM) buffer or DMEM (cells) and different combinations of substrates and inhibitors were added before CoQ extraction and LC-MS/MS analysis of the CoQ redox state. All incubations were performed for 5 min at 37°C except otherwise indicated. CoQ redox state of: **c** BHMM incubated with NADH or NADH combined with rotenone or KCN. **d** BHMM incubated with succinate or succinate combined with malonate or KCN. **e** BHMM incubated for 20 min at 37°C. NADH or rotenone + NADH was added to indicated samples after 15 min **f** RHM incubated with glutamate + malate (GM) or GM combined with rotenone or KCN. **g** RHM incubated with succinate or succinate combined with malonate, FCCP or KCN. **h** C2C12 cells incubated in standard DMEM with FCCP, rotenone, TTFA or Antimycin A. For all experiments, data are represented as mean ± S.D. of 3 replicates. The proportion of CoQH_2_ is shown for the indicated most prevalent CoQ species.

To assess the efficacy of this procedure we measured the CoQ redox state in bovine mitochondrial membranes, which contain predominantly CoQ_10_, respiring on NADH or succinate in the presence of various inhibitors (Fig. 5b). Membranes respiring on NADH or succinate had a CoQ pool of about 56–60% reduced. With NADH the complex I inhibitor rotenone decreased this to ~4% reduced (Fig. 5c), while with succinate the complex II inhibitor malonate also maintained an oxidised CoQ pool of ~12% reduced (Fig. 5d). Addition of the complex IV inhibitor KCN led the CoQ pool to be ~80% reduced (Fig. 5c and d). Incubating membranes at 37°C in the absence of substrates led to complete oxidation of the CoQ pool, which can subsequently be reduced to ~60% by NADH (Fig. 5e).

We next examined isolated rat heart mitochondria, which contain mainly CoQ_9_. Mitochondria respiring on glutamate/malate to generate NADH led to reduction of the CoQ pool (Fig. 5f). The CoQ pool was oxidised by blocking complex I with rotenone and increased to ~77% reduction upon addition of KCN. Using succinate as a substrate also led to CoQ reduction that was decreased by malonate and increased by KCN. Addition of the uncoupler FCCP to abolish Δp and thereby stimulate respiration oxidised the CoQ pool (Fig. 5g).

To assess CoQ in a monolayer of C2C12 cells in culture we first rapidly washed the cell layer and then scraped the cells into cold PBS before extraction. As C2C12 cells are derived from mice, CoQ_9_ is the predominant form of CoQ. Cells showed a similar steady state CoQ redox state to isolated mitochondria, and this was oxidised by blocking complexes I and II with rotenone and TTFA, respectively (Fig. 5h). Uncoupling mitochondria with FCCP oxidises the CoQ pool due to increased respiration, and blocking complex III with antimycin led to reduction of the CoQ pool (Fig. 5h).

In all three systems, there was a dominant CoQ species, either CoQ_9_ or CoQ_10_, along with a far smaller amount of the other form. The redox state of the major form is reported above, but the method detected both forms accurately. In all cases the redox state of the major and minor CoQ species were the same, suggesting that both pools come to the same redox state (Figs. S3a–f).

### *2.5* *Measurement of the CoQ redox state in vivo*

**Fig. 6:**
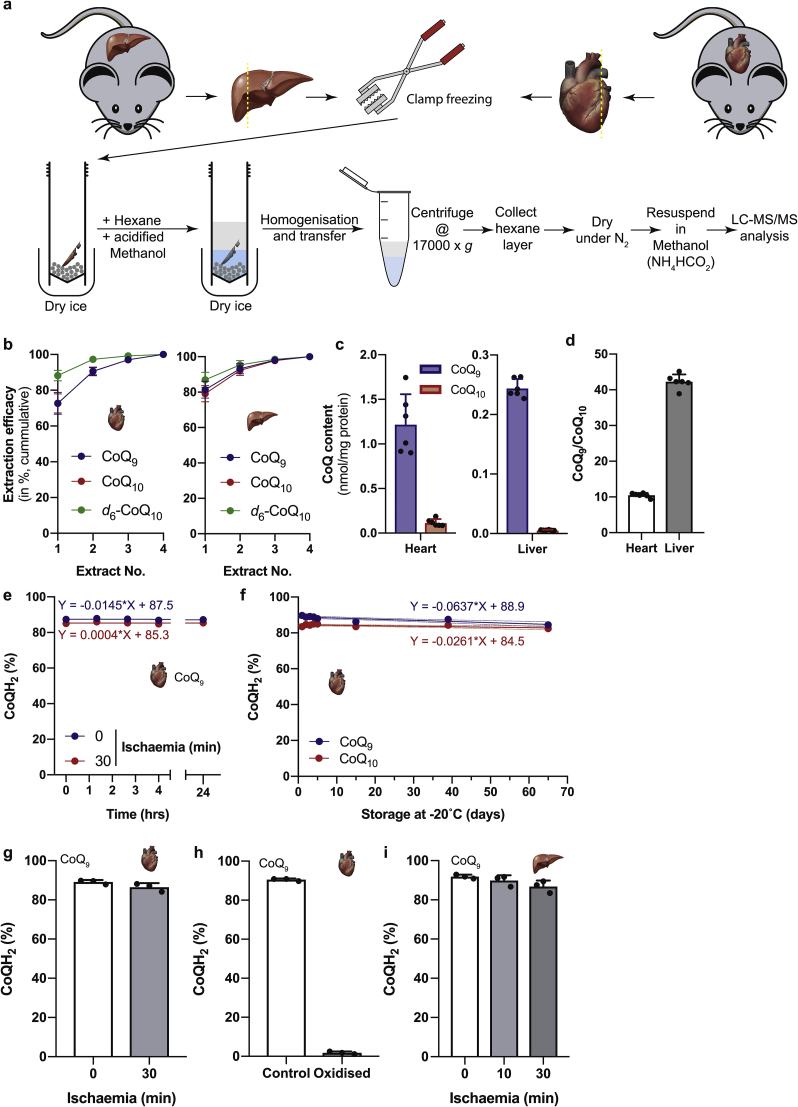
Extracting CoQ from tissues and determining the CoQ redox state. **a** A two-phase extraction with acidified methanol and hexane was used to extract CoQ from tissues. CoQ from clamp frozen tissue samples was extracted by homogenising in extraction solution, phase separation by centrifugation and resuspending the extract in MS sample buffer (methanol with 2 mM ammonium formate). **b** CoQ was extracted from mouse heart and liver tissue homogenates and the tissue homogenate was then reextracted. The peak areas for CoQ and CoQH_2_ were combined and the cumulative proportion combined with all previous extractions of the total is shown. Data are shown as mean ± S.D. of 6 different samples from 3 different animals. **c** The CoQ pool size of CoQ_9_ and CoQ_10_ in mouse heart and liver. Data are shown as mean ± S.D. of 6 different samples from 3 different animals. **d** The CoQ_9_/CoQ_10_ ratio in mouse heart and liver. Data are shown as mean ± S.D. of 6 different samples from 3 different animals. **e** CoQ redox state changes over time (at 8°C) during several consecutive LC-MS runs (~80 min per run per sample set; final run at 24 h) in CoQ extracts of control and ischaemic mouse hearts. The proportion of CoQH_2_ is represented as mean ± S.D. of 3 different hearts. **f** CoQ redox state changes during long term storage for up to 65 days (at −20°C) in CoQ extracts of control mouse hearts. The proportion of CoQH_2_ is represented as mean ± S.D. of 3 different hearts. **g** CoQ redox state of control and ischaemic mouse heart. The proportion of CoQH_2_ is represented as mean ± S.D. of 3 different hearts. **h** CoQ redox state of mouse heart tissue and tissue homogenates of the same hearts, after oxidising the CoQ pool in KPi buffer for 1 h at 37°C. The proportion of CoQH_2_ is represented as mean ± S.D. of 3 different hearts. **i** CoQ redox state of control and ischaemic (10 and 30 min) mouse liver. The proportion of CoQH_2_ is represented as mean ± S.D. of 3 different livers.

We next extended the two-phase extraction to assess CoQ redox state *in vivo*. To do this we focussed on the mouse heart and liver as these are important tissues in which the CoQ redox state is important in health and disease. In assessing tissues, it is vital to freeze the tissue as rapidly as possible so that the measured CoQ redox state accurately reflects that *in vivo*. To illustrate this, heart or liver tissue was excised either from terminally anaesthetized mice after thoracotomy, or after cervical dislocation and then rapidly frozen in a Wollenberger clamp at liquid nitrogen temperature. The frozen tissue was then homogenised in ice-cold acidified methanol and hexane and analysed by LC-MS/MS (Fig. 6a).

We first determined how well this procedure extracted CoQ from the tissue (Figs. 6b, S4a,b). This was done by re-extracting the heart and liver tissue and seeing how much residual CoQ was extracted (Figs. 6b, S4a,b). This showed that the first extraction removed ~75–80% of the CoQ pool. While subsequent extractions could remove a bit more, it was decided not to include a second extraction routinely.

We next quantified the CoQ pool in heart and liver and found ~1.2 nmol CoQ_9_ and ~0.12 nmol CoQ_10_ per mg protein in heart (Fig. 6c). In the liver the CoQ pools are much smaller with ~250 pmol CoQ_9_ and ~6 pmol CoQ_10_ per mg protein (Fig. 6c). These values are consistent with the previously reported values [25,26]. Interestingly the CoQ_9_/CoQ_10_ ratio varied significantly between organs, being ~10 in heart and ~42 in liver (Fig. 6d).

An important constraint in analysing the CoQ redox state is the oxidation of extracts by air before analysis. This often requires the rapid analysis of sample which are processed in small batches. To see if this was a concern, we reanalysed the CoQ redox state of samples stored in the LC-MS/MS autosampler at 8°C for up to 24 h in argon-flushed tubes. The CoQ redox state was stable for at least 24 h in the autosampler, confirming that large sample sets can be analysed in one run without artefactual oxidation (Figs. 6e and S4c). The CoQ redox state was stable without argon flushing (Fig. S4d). Nevertheless, we continued to flush samples with argon as a precaution. We next investigated the stability of the CoQ redox state of extracts during long-term storage at −20°C in argon-flushed tubes. This showed that the CoQ redox state was stable upon storage for up to at least 65 days (Fig. 6f). This allows to store and combine separate CoQ extractions in a single analysis run.

We noticed that CoQ extracts from mouse hearts were far more reduced than previously reported values (Fig. 6e) [18]. Consequently, we wanted to assess whether there was any artefactual reduction of CoQ during extraction. To do this we set up a detection protocol for *d*_6_-CoQ_10_ and *d*_6_-CoQ_10_H_2_ as described in section 2.1 (Figs. S4e and f). We then measured the reduction of *d*_6_-CoQ_10_ when it was spiked into the tissue extraction solution and detected ~6% of the added *d*_6_-CoQ_10_ as *d*_6_-CoQ_10_H_2_ in our extracts (Fig. S4g). In a mock extraction without tissue ~0.8% of the added *d*_6_-CoQ_10_ was converted to *d*_6_-CoQ_10_H_2_. No signal for *d*_6_-CoQ_10_ or *d*_6_-CoQ_10_H_2_ was detected in unspiked tissue extracts (Fig. S4g). Therefore, we conclude that a small amount of CoQ reduction can occur during extraction. However, completely oxidised d_6_-CoQ_10_ is thermodynamically much more prone to reduction than the already largely reduced CoQ pools found *in vivo* and even if the oxidised CoQ pool was underestimated by 5% this would have a minor effect on the overall redox state.

Next, we wanted to determine the CoQ redox state in clamp frozen heart. In mice killed by cervical dislocation the CoQ pool was highly reduced at 89% and remained at a similar redox state even after 30 min of warm ischaemia (Figs. 6g and S5a). To explore whether the tissue collection time from cervical dislocation to clamp-freezing has an effect on CoQ redox state, we next assessed the level of CoQ redox state in hearts obtained from terminally anaesthetized mice. In these mice the heart was excised directly after chest opening. This enabled a still-beating heart to be rapidly frozen and clamped. This gave a CoQH_2_% reduction of 85% (Fig. S5b), similar to that from hearts excised from animals after cervical dislocation. The redox state of the CoQ_10_ pool was also similar indicating that, as mentioned above, the two CoQ pools are bioenergetically equivalent (Figs. 6g, S5a,b). The CoQ pool can be fully oxidised by homogenising the heart in buffer and incubation at 37°C for 1 h (Figs. 6h and S5c). We also determined the CoQ redox state in freshly excised and ischaemic liver and found it to be highly reduced at 92% (Figs. 6i and S5d). As in the heart we also did not observe any significant changes in the CoQ redox state during ischaemia in liver (Figs. 6i and S5d). For all tested tissues the LC-MS/MS traces were clean and the retention times of the individual CoQ species matched those of pure CoQ (Fig. S5e).

## 3 Conclusions

Changes in the CoQ redox state have broad implications in the regulation of metabolic processes, respiration and mitochondrial ROS production. The difficulty in determining the CoQ redox state in biological samples and *in vivo* have been a significant impediment for the characterisation of CoQ function within mitochondria under physiological and pathophysiological conditions. Here we have developed a sensitive and comparable approach to assess the CoQ redox state by LC-MS in isolated mitochondria, cells and tissues. Applying this approach, we are able to detect CoQ and CoQH_2_ at equal sensitivity, allowing relative quantification without the need for a range of internal standards, an improvement on previous methods, which used standard curves for both CoQ and CoQH_2_. In parallel, the total CoQ pool size can be determined using only a standard curve of oxidised CoQ. In contrast to related approaches [18], we found that while the redox states of CoQ_9_ and CoQ_10_ can be manipulated with inhibitors and uncouplers they were essentially the same during normal conditions in a range of systems. Furthermore, we found that the CoQ redox state is highly (~90%) reduced in tissues *in vivo*, something that was not found in other studies, perhaps due to oxidation of the CoQ pool during extraction and processing [18]. Thus our method enables the redox state of the CoQ pool *in vivo* to be determined more accurately than hitherto. Our protocol opens up new possibilities to unravel the role of the CoQ redox state *in vivo* to understand the interplay between metabolism and bioenergetics in physiological and pathophysiological processes.

## 4 Materials and methods

### 4.1 Materials

All chemicals were purchased from Sigma unless otherwise stated. LC-MS grade Methanol was purchased from Fisher. All buffers, except LC-MS buffers, were stored over Chelex-100. Acidified solvents were prepared by addition of 0.1% (w/v) HCl.

### 4.2 Synthesis of d_6_-CoQ_10_

Sodium hydroxide (400 μL, 1 M in CD_3_OD) was added to a solution of CoQ_10_ (400 mg, 0.46 mmol, 1.0 eq) in hexane (5 mL) and *d*_*4*_-MeOD (4 mL). The solution was stirred at RT for 6 h then quenched with acetic acid. The solution was extracted into diethyl ether (~20 mL), washed with brine (2 × 75 mL), dried over sodium sulfate and concentrated under vacuum. The residue was purified by automated column chromatography using a 25g SNAP ultra cartridge on a Biotage Isolera using gradient elution from CH_2_Cl_2_:Hexane (0:100) to (80:20). The product was recrystallised from isopropanol-diethyl ether to give the labelled CoQ_10_ as an orange solid (162 mg, 40%). *δ*_H_ (400 MHz: CDCl_3_): 5.13–5.04 (9H, m, 9 × CH = ), 4.94 (1H, tq, *J* = 7.1 + 1.4 Hz, CH = ), 3.18 (2H, d, *J* = 7.0 Hz, CH_2_), 2.10–2.03 (16H, m, 8 × CH_2_), 2.01 (3H, s, CH_3_), 2.00–1.93 (20H, m, 10 × CH_2_), 1.74 (3H, s, CH_3_), 1.68 (3H, s, CH_3_), 1.60 (21H, broad s, 7 × CH_3_), 1.58 (3H, s, CH_3_). m/z (ESI): Found: 891.7120. C_59_H_84_D_6_NaO_4_ requires (M + Na) ^+^, 891.7108.

### 4.3 Calculation of CoQ reduction potential

The CoQ reduction potential was calculated using the following equation, assuming a matrix pH of 7.7 [24]. E_h_ for the CoQ/CoQH_2_ couple in mV is:

$$E_{h}\left(\frac{CoQ}{CoQH_{2}}\right)=-38+30.5log_{10}\left(\frac{CoQ}{CoQH_{2}}\right)$$

### 4.4 Preparation of mitochondria and mitochondrial membranes

Bovine heart mitochondrial membranes were kindly provided by Dr Hiran Prag and Prof Judy Hirst (MRC MBU) and were prepared as described previously [27]. Female Wistar rats (Charles River) of 10–12 weeks of age were killed by stunning, followed by cervical dislocation. Rat hearts were homogenised in STEB (250 mM sucrose, 5 mM Tris-HCl, 1 mM EGTA, 0.1% fatty acid free BSA, pH 7.4, 4°C). Mitochondria were isolated by differential centrifugation (2x 700×*g* for 5 min, 2 × 5,500×*g* for 10 min) at 4°C and the protein content was determined by the bicinchoninic acid assay using the Pierce™ BCA Protein assay kit with bovine serum albumin as a standard.

### 4.5 Mouse heart and liver experiments

All mouse experiments were carried out in accordance with the UK Animals (Scientific Procedures) Act 1986 and the University of Cambridge Animal Welfare policy (Project license 70/8238). Male mice (8–10 weeks; C57BL/6J; Charles River Laboratories) were terminally anaesthetized with sodium pentobarbital injected intraperitoneally and ventilated with O_2_. Core temperature was monitored continuously via a rectal probe. Alternatively, mice were culled by cervical dislocation. A sternal thoracotomy was performed (heart) or the abdomen was opened (liver) and the organ was rapidly excised and then frozen with a Wollenberger clamp cooled in liquid nitrogen followed by storage at −70°C. Ischaemia was induced by excising the organ and keeping it in the abdomen or chest of the warmed mouse for indicated lengths. Organs were then frozen and stored as described.

### 4.6 Reduction of ubiquinone

Ubiquinones (CoQ_9_, CoQ_10_ and *d*_6_-CoQ_10_) were reduced to ubiquinols using sodium borohydride. All glassware was rinsed in hexane and dried under nitrogen before use. CoQ_9_ or CoQ_10_ were made up to 10 mM in hexane, then diluted 1:10 in hexane. Solid sodium borohydride was added (2 mg/100 μl) to the CoQ dilution followed by addition of methanol (to 5% v/v), vortexing for 3 min and incubation in the dark for 5 min. The reaction was stopped by addition of 1 vol acidified H_2_O with vortexing for 1 min followed by centrifugation (1,500×*g*, 5 min at 4°C). The CoQ-containing upper hexane layer was transferred to a glass vial, overlaid with argon and stored at −20°C. UV scanning spectra confirmed reduction and enabled the concentration to be calculated: ε_290_ = 4.1 mM^−1^cm^−1^ (CoQ_9_H_2_), 4.0 mM^−1^cm^−1^ (CoQ_10_H_2_); ε_275_ = 14.7 mM^−1^cm^−1^ (CoQ_9_), 14.6 mM^−1^cm^−1^ (CoQ_10_) [28].

### 4.7 Mass spectrometry

The mass spectrometric fragmentation patterns for the ammonium adducts of each CoQ CoQH_2_ compound were determined by direct infusion of 10 μM of the compound in 2 mM ammonium formate in methanol at 2 μl/min into a triple quadrupole mass spectrometer (Waters Xevo TQ-S). Electrospray ionisation in positive ion mode was used as published previously with the following settings [29]: capillary voltage – 1.7 kV; cone voltage – 30 V; ion source temperature – 100°C; collision energy – 22 V. Nitrogen and argon were used as the curtain and the collision gases, respectively.

### 4.8 Liquid chromatography tandem mass spectrometry

LC-MS/MS analyses were carried out using an I-Class Acquity LC system attached to a Xevo TQ-S triple quadrupole mass spectrometer (Waters) using published parameters [29]. Samples were kept at 8°C prior to injection by the autosampler of 2–10 μl into a 15 μl flow-through needle and RP-HPLC at 45°C using an Acquity C18 column (2.1 × 50 mm, 1.7 μM; Waters). Mobile phase was isocratic 2 mM ammonium formate in methanol run at 0.8 ml/min over 5 min. For MS analysis, electrospray ionisation in positive ion mode was used as described above. Multiple reaction monitoring in positive ion mode was used for compound detection. Transitions used for quantification were: CoQ_9_, 812.9 > 197.2; CoQ_10_, 880.9 > 197.2; CoQ_9_H_2_, 814.9 > 197.2; CoQ_10_H_2_, 882.9 > 197.2; *d*_*6*_-CoQ_10_, 887.0 > 203.1; *d*_*6*_-CoQ_10_H_2_, 889.0 > 203.1. Standard curves were prepared using known amounts of CoQ, which were spiked with d_6_-CoQ_10_ (IS). Standards and samples were quantified using MassLynx 4.1 software to determine the peak area for CoQ_9_, CoQ_10_, CoQ_9_H_2_, CoQ_10_H_2_, *d*_*6*_-CoQ_10_ and *d*_*6*_-CoQ_10_H_2_, and the standard curves used to determine the total amount of CoQ present in samples.

### 4.9 Mitochondrial incubations

Mitochondrial membrane or rat heart mitochondria were incubated at 15 μg protein in 100 μl KPi buffer (50 mM KPi, pH 7.8) or KCl buffer (120 mM KCl, 10 mM HEPES, 1 mM EGTA. pH 7.2), respectively. Substrates and inhibitors were added at the following concentrations where indicated: 1 mM NADH, 1 mM succinate, 1 mM glutamate/malate, 0.5 μM carbonyl cyanide-*p*-trifluoromethoxyphenylhydrazone (FCCP), 0.5 μM rotenone, 1 mM malonate, 1 mM KCN. Samples were incubated with shaking for 5 min or 20 min at 37°C.

### 4.10 Cell incubations

C2C12 mouse myoblast cells (ATCC, UK) were cultured at 37°C under a humidified atmosphere of 5% CO_2_ in Dulbecco's modified Eagles's medium (DMEM/4.5 g/l glucose + GlutaMAX, Gibco, UK) supplemented with 10% fetal bovine serum, 100 units/ml penicillin and 100 mg/ml streptomycin. Cells (100,000) were plated 24 h prior to experiment in 6-well plates with 2 ml medium. For experiments inhibitors were added at the following concentrations: 1 μM rotenone, 1 mM theonoyltrifluoroacetone (TTFA), 5 μM FCCP, 5 μM Antimycin A. Cells were then incubated for 5 min at 37°C, washed with PBS and scraped in 200 μl fresh PBS on ice prior to CoQ extraction.

### 4.11 CoQ extraction

To extract CoQ from mitochondrial membranes and mitochondria, incubations of 100 μl containing 15 μg protein were added to ice cold extraction solution (200 μl acidified methanol and 200 μl hexane) followed by vortexing. To extract CoQ from cells, the cell suspension in 200 μl PBS was transferred to ice cold extraction solution (200 μl acidified methanol and 300 μl of hexane) followed by vortexing. To extract CoQ from tissues, 5 mg frozen tissue was weighed into cooled lysis tubes (Precellys, CK-14) on dry ice. Then a mixture of 250 μl ice-cold acidified methanol and 250 μl hexane was added, and tissue was homogenised in the bullet blender (Bertin Instruments, Precellys 24) at setting 6500 during 15 s (CK14 (1.4 mm) beads). The homogenate was transferred to fresh 1.5 ml Eppendorf tubes. In all cases the upper, CoQ-containing hexane layer was separated by centrifugation (5 min, 17,000×*g*, 4°C) and then dried down in 1 ml glass mass spectrometry vials (186005663CV, Waters) under a stream of N_2_. Dried samples were then resuspended in methanol containing 2 mM ammonium formate and diluted (heart 1:10, liver not dilution: CoQ_10_ or 1:3 dilution CoQ_9_), overlaid with argon and stored at −20°C until analysis. The extracts were stable under storage for up to at least 15 days.

### 4.12 CoQ extraction for CoQ pool size determination

To extract CoQ from tissues, 5 mg frozen tissue was weighed into cooled lysis tubes (Precellys, CK-14) on dry ice. Then 150 μl KPi buffer (50 mM KPi, pH 7.8) was added and tissue was homogenised in the bullet blender (Bertin Instruments, Precellys 24) at setting 6500 during 15 s (CK14 (1.4 mm) beads). CoQ was extracted from 100 μl of the homogenate by adding a mixture of 250 μl ice-cold acidified methanol and 250 μl hexane and vortexing. The upper, CoQ-containing hexane layer was separated by centrifugation (5 min, 17,000×*g*, 4°C) and then dried down in 1 ml glass mass spectrometry vials (186005663CV, Waters) under a stream of N_2_. Dried samples were then resuspended in methanol containing 2 mM ammonium formate. Heart tissue was diluted 1:10 (20 μl in 170 μl methanol containing 2 mM ammonium formate and 10 μl of 5 μM d_6_-CoQ_10_ internal standard), while liver homogenate was either not diluted (CoQ_10_ analysis) or diluted 1:3 (CoQ_9_ analysis). Samples were overlaid with argon and stored at −20°C until analysis.

## Figures and Tables

**figs1:**
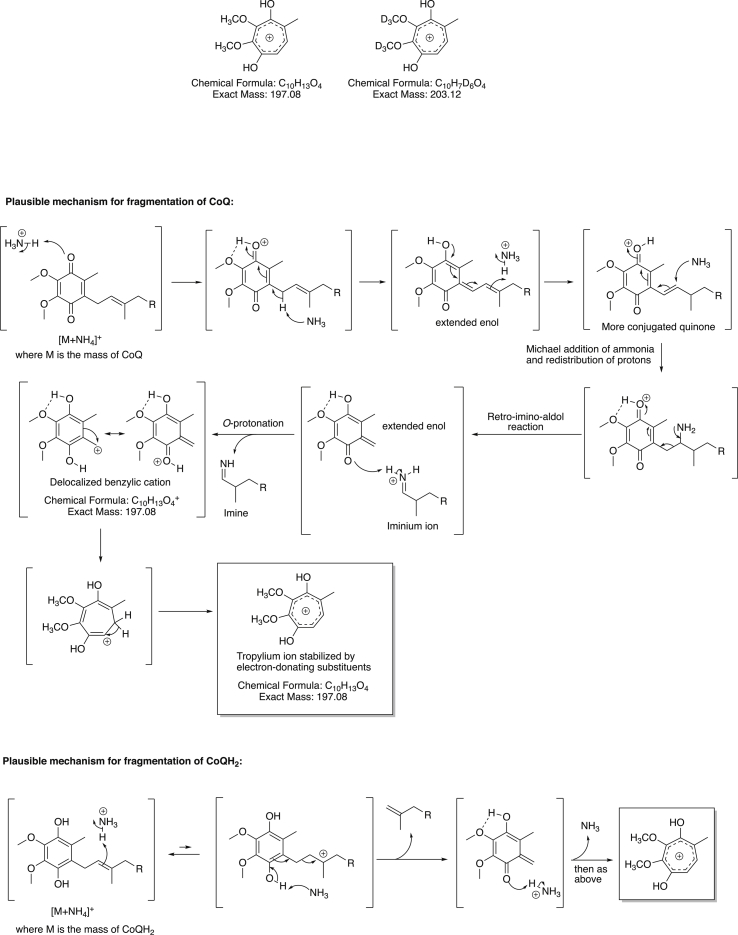


**figs2:**
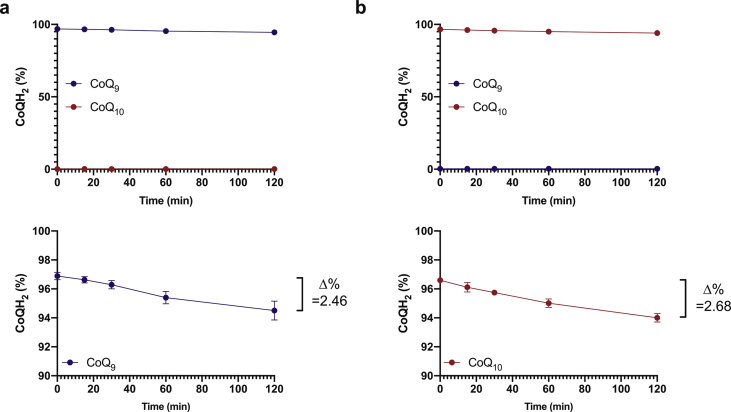


**figs3:**
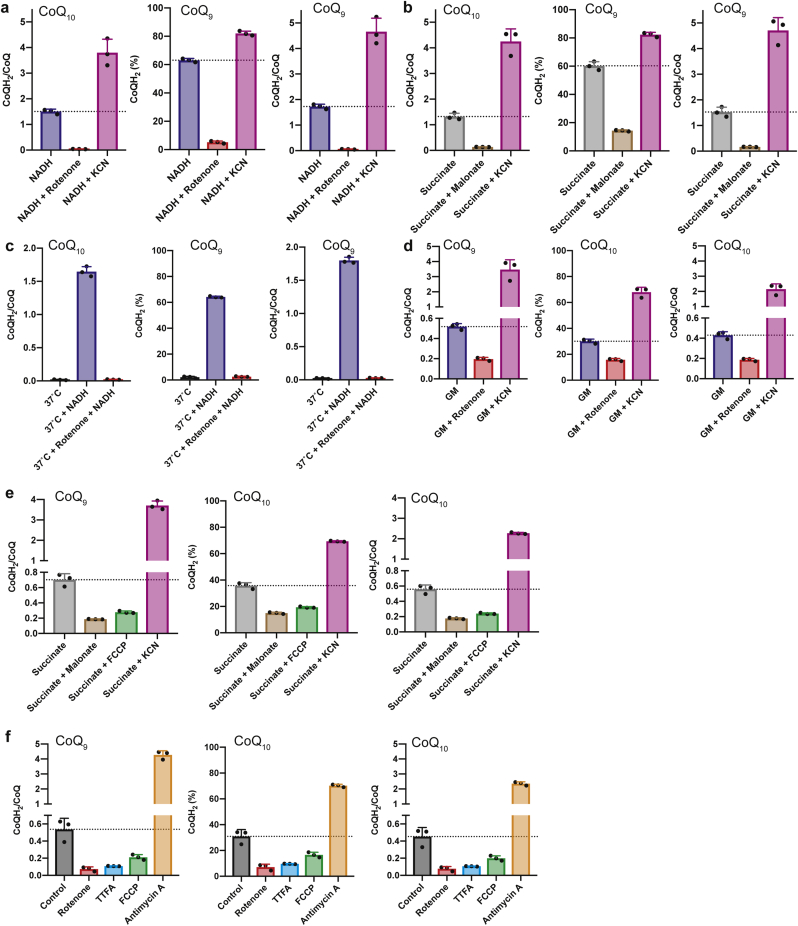


**figs4:**
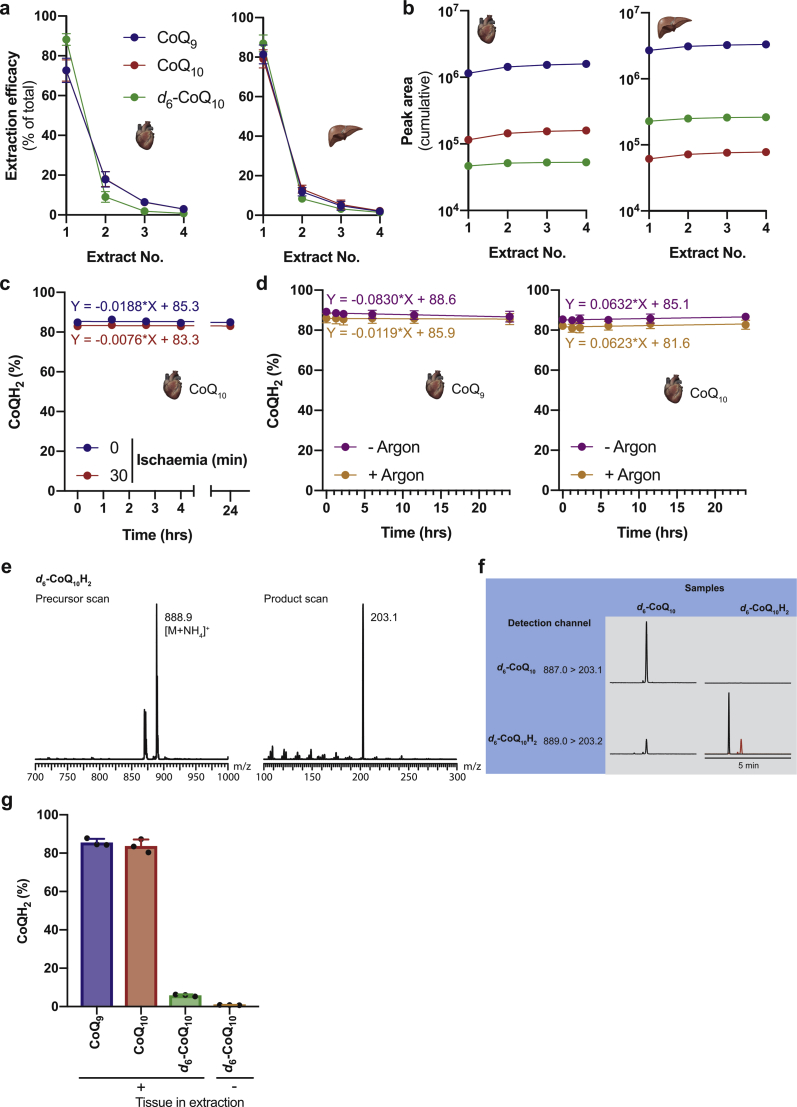


**figs5:**
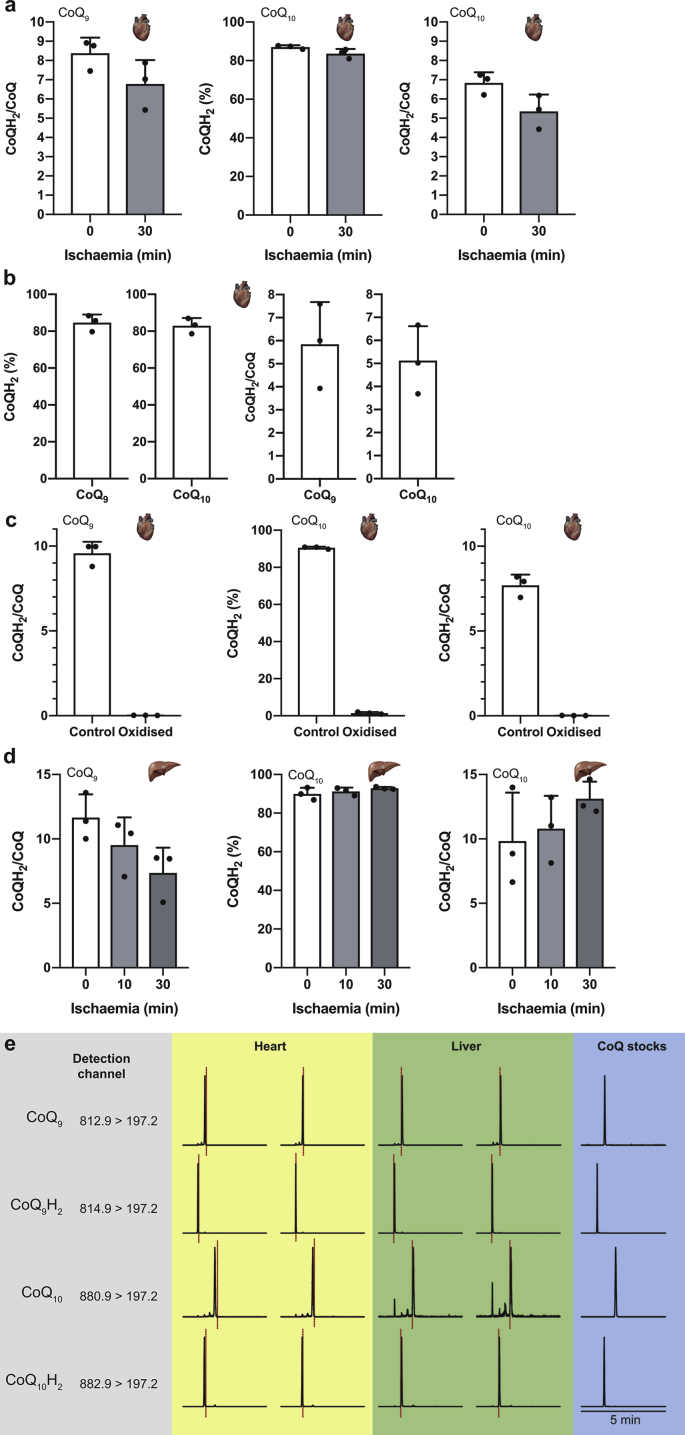


## Abbreviations

Δp: mitochondrial protonmotive force
CoQ_10_: coenzyme Q_10_
CoQ_10_H_2_: reduced Q_10_
CoQ_9_: coenzyme Q_9_
CoQ_9_H_2_: reduced Q_9_
ETC: electron transfer chain
IS: internal standard
LC-MS/MS: liquid chromatography tandem mass spectrometry
MRM: multiple reaction monitoring
RET: reverse electron transfer
ROS: reactive oxygen species
