# Supplementary material for: A sensitive mass spectrometric assay for mitochondrial CoQ pool redox state in vivo
Source: Free Radic Biol Med. 2020 Feb 1;147:37–47. doi: 10.1016/j.freeradbiomed.2019.11.028 (PMC6975167; doi:10.1016/j.freeradbiomed.2019.11.028)
Supplement: Multimedia component 1 [file mmc1.docx]

**A sensitive mass spectrometric assay for mitochondrial CoQ pool redox state *in vivo***

Nils Burger^a^, Angela Logan^a^, Tracy A. Prime^a^, Amin Mottahedin^a,b,c^, Stuart T. Caldwell^d^, Thomas Krieg^b^, Richard C. Hartley^d^, Andrew M. James^a^, Michael P. Murphy^a, b^*

^a^MRC Mitochondrial Biology Unit, University of Cambridge, Hills Road, Cambridge CB2 0XY, UK

^b^Department of Medicine, University of Cambridge, Addenbrooke’s Hospital, Cambridge CB2 0QQ, UK

^c^Department of Physiology, Institute of Neuroscience and Physiology, Sahlgrenska Academy, University of Gothenburg, Gothenburg, Sweden

^d^School of Chemistry, University of Glasgow, Glasgow G12 8QQ, UK

*Corresponding author

Prof Michael P. Murphy: mpm@mrc-mbu.cam.ac.uk

Phone: +44 1223 252900

**SUPPLEMENTARY FIGURE LEGENDS**

*Supplementary Figure 1: Proposed mechanism for fragmentation of CoQ and CoQH_2_ ammonium adducts*

CoQ and CoQH_2_ ammonium adducts are fragmented as indicated within the MS upon collision with argon. This fragmentation of both CoQ and CoQH_2_ leads to the same product ion with an m/z 197. The extended enol could lead to a range of possible ions with this m/z including a protonated quinone (by C-protonation, not shown), the delocalized benzylic cation [1,2], or a substituted tropylium ion [3,4]. The tropylium ion substituted with electron-donating groups is likely to be the most stable structure, and can be formed by rearrangement of the delocalized benzylic cation [5].

*Supplementary Figure 2: In methanol no interspecies exchange between CoQH_2_ and CoQ is detectable*

To determine if interspecies CoQ electron transfer can occur in methanol, CoQ_9_H_2_/CoQ_10_ (**a**) and CoQ_10_H_2_/CoQ_9_ (**b**) were combined at equal concentrations and the CoQ redox state was determined by LC-MS/MS for different time points at 37˚C for up to 2 hours. The proportion of oxidation of the reduced CoQ species over two hours is indicated. Data are represented as mean ± S.D. of 4 replicates.

*Supplementary Figure 3: Modifying the CoQ redox state in mitochondrial membranes, mitochondria and cells*

**a-f** Bovine heart mitochondrial membranes (BHMM), rat heart mitochondria (RHM) and C2C12 cells were incubated in KPi (BHMM), KCl (RHM) buffer or DMEM (cells) and different combinations of substrates and inhibitors were added before CoQ extraction and LC-MS/MS analysis of the CoQ redox state. All incubations were performed for 5 minutes at 37˚C except otherwise indicated. CoQ redox state of: **a** BHMM incubated with NADH or NADH combined with rotenone or KCN. **b** BHMM incubated with succinate or succinate combined with malonate or KCN. **c** BHMM incubated for 20 min at 37˚C. NADH or rotenone + NADH was added to indicated samples after 15 minutes. **d** RHM incubated with glutamate + malate (GM) or GM combined with rotenone or KCN. **e** RHM incubated with succinate or succinate combined with malonate, FCCP or KCN. **f** C2C12 cells incubated in standard DMEM with FCCP, rotenone, TTFA or Antimycin A.

For all experiments, data are represented as mean ± S.D. of 3 replicates. The proportion of CoQH_2_ or the CoQH_2_/CoQ ratios are shown for the indicated CoQ species.

*Supplementary Figure 4: Extracting CoQ from tissues and determining the CoQ redox state*

**a** CoQ was extracted from mouse heart and liver tissue homogenates and the tissue homogenate was then reextracted. The peak areas for CoQ and CoQH_2_ were combined and the proportion per extraction of the total is shown. Data are shown as mean ± S.D. of 6 different samples from 3 different animals. **b** CoQ was extracted from mouse heart and liver tissue homogenates and the tissue homogenate was then reextracted. The detected peak areas for CoQ and CoQH_2_ are depicted cumulatively. Data are shown as mean ± S.D. of 6 different samples from 3 different animals. **c** CoQ redox state changes over time (at 8˚C) during several consecutive LC-MS runs (~80 min per run per sample set; final run at 24 hours) in CoQ extracts of control and ischaemic mouse hearts. The proportion of CoQH_2_ is represented as mean ± S.D. of 3 different hearts. **d** CoQ redox state changes over time (at 8˚C) during several consecutive LC-MS runs (final run at 24 hours) in CoQ extracts of control mouse hearts +/- argon overlay. CoQ extracts were dried under N_2_ and resuspended in methanol containing ammonium formate and overlaid with argon for resuspension. CoQ extracts were then split into two +/- argon overlay for analysis. The proportion of CoQH_2_ is represented as mean ± S.D. of 3 different hearts. **e** Representative precursor and product MS scans of *d*_6_-CoQ_10_H_2_. The precursor scan of the characteristic fragment product was performed. The product scan was performed by fragmenting the ammonium adduct precursor. **f** Representative LC-MS/MS chromatograms showing the m/z transitions measured simultaneously for 0.5 pmol of *d*_6_-CoQ_10_ and *d*_6_-CoQ_10_H_2_. Traces are normalised to the highest peak for each sample. d_6_-CoQ_10_ shows bleed through into the transition for *d*_6_-CoQ_10_H_2_. Bleed through of the *d*_6_-CoQ_10_ signal has been projected in red onto the *d*_6_-CoQ_10_H_2_ trace to show they are separated by LC. **g** CoQ redox state of control mouse heart and oxidised *d*_6_-CoQ_10_ which was spiked into the extraction solution. A mock extraction with only oxidised *d*_6_-CoQ_10_ in the absence of tissue was additionally performed. No bleed through into the *d*_6_-CoQ_10_ and *d*_6_-CoQ_10_H_2_ channels was detected in plain heart extracts. The proportion of CoQH_2_ is represented as mean ± S.D. of 3 different hearts.

*Supplementary Figure 5: Extracting CoQ from tissues and determining the CoQ redox state*

**a** CoQ redox state of control and ischaemic mouse heart. The proportion of CoQH_2_ and the CoQH_2_/CoQ ratios are represented as mean ± S.D. of 3 different hearts. **b** CoQ redox state of control mouse heart excised from terminally anaesthetised animals after thoracotomy. The proportion of CoQH_2_ and the CoQH_2_/CoQ ratios are represented as mean ± S.D. of 3 different hearts. **c** CoQ redox state of mouse heart tissue and tissue homogenates of the same hearts, after oxidising the CoQ pool in KPi buffer for 1 hr at 37˚C. The proportion of CoQH_2_ and the CoQH_2_/CoQ ratios are represented as mean ± S.D. of 3 different hearts. **d** CoQ redox state of control and ischaemic (10 and 30 min) mouse liver. The proportion of CoQH_2_ and the CoQH_2_/CoQ ratios are represented as mean ± S.D. of 3 different livers. **e** Representative LC-MS/MS chromatograms showing the m/z transitions measured simultaneously for heart and liver extracts and 0.5 pmol of CoQ_9_, CoQ_10_, CoQ_9_H_2_ and CoQ_10_H_2_. Traces are normalised to the highest peak for each sample. The retention time for the individual CoQ and CoQH_2_ stocks is indicated in the tissue traces by a red line.

For all experiments, values are shown for the indicated CoQ species.

**References**

[1] B. Marbois, P. Gin, K.F. Faull, W.W. Poon, P.T. Lee, J. Strahan, J.N. Shepherd, C.F. Clarke, Coq3 and Coq4 define a polypeptide complex in yeast mitochondria for the biosynthesis of coenzyme Q., J. Biol. Chem. 280 (2005) 20231–20238. doi:10.1074/jbc.M501315200.

[2] R.F. Muraca, J.S. Whittick, G.D. Daves, P. Friis, K. Folkers, Mass Spectra of Ubiquinones and Ubiquinols, J. Am. Chem. Soc. 89 (1967) 1505–1508. doi:10.1021/ja00982a038.

[3] B.C. Brajcich, A.L. Iarocci, L.A.G. Johnstone, R.K. Morgan, Z.T. Lonjers, M.J. Hotchko, J.D. Muhs, A. Kieffer, B.J. Reynolds, S.M. Mandel, B.N. Marbois, C.F. Clarke, J.N. Shepherd, Evidence that ubiquinone is a required intermediate for rhodoquinone biosynthesis in Rhodospirillum rubrum., J. Bacteriol. 192 (2010) 436–445. doi:10.1128/JB.01040-09.

[4] T.P.T. Nguyen, A. Casarin, M.A. Desbats, M. Doimo, E. Trevisson, C. Santos-Ocaña, P. Navas, C.F. Clarke, L. Salviati, Molecular characterization of the human COQ5 C-methyltransferase in coenzyme Q10 biosynthesis, Biochim. Biophys. Acta - Mol. Cell Biol. Lipids. 1841 (2014) 1628–1638. doi:10.1016/J.BBALIP.2014.08.007.

[5] T.D. Fridgen, J. Troe, A.A. Viggiano, A.J. Midey, S. Williams, T.B. McMahon, Experimental and theoretical studies of the benzylium +/ Tropylium + ratios after charge transfer to ethylbenzene, J. Phys. Chem. A. 108 (2004) 5600–5609. doi:10.1021/jp031328s.
